# Recent advances in understanding and managing asthma

**DOI:** 10.12688/f1000research.9236.1

**Published:** 2016-08-23

**Authors:** Su-Ling Loo, Peter A.B. Wark

**Affiliations:** 1 Priority Research Centre for Healthy Lungs, University of Newcastle, Hunter Medical Research Institute, New Lambton, NSW, 2305, Australia

**Keywords:** asthma, childhood asthma, understanding and treatment of asthma

## Abstract

This review highlights the important articles published in the area of asthma research from January 2015 to July 2016. In basic science, significant advances have been made in understanding the link between the innate immune response and type II acquired immune responses in asthma and the role of the airway epithelium. Novel information continues to emerge with regard to the pathogenesis and heterogeneity of severe asthma. There have been important translational clinical trials in the areas of childhood asthma, treatment of allergy to improve asthma outcomes, and improving drug delivery to optimize the management of asthma. In addition, there are increasing data concerning the application of biological agents to the management of severe asthma. This body of work discusses the most notable advances in the understanding and management of asthma.

## Introduction

In recent years, there have been major advances in the understanding and treatment of asthma. A greater understanding of the mechanisms of disease through basic science research over the last twenty years has resulted in the development of highly specific treatments that better target the dysregulated immune processes behind asthma and now are being translated into new effective disease-modifying treatments. At the same time, translational clinical research has opened our eyes to the diverse biology of asthma and is starting to lead investigators towards mechanisms that are not confined to allergic and type II immune responses to meet the needs of greater numbers of asthma sufferers. These changes have made for an exciting time in asthma research, with a large increase in novel interventions going into clinical trials. This article will review selected important articles that have been published in the literature from January 2015 to July 2016 in the fields of basic science leading to better understanding of the mechanisms behind asthma, as well as important or potentially important clinical trials that may transform its management.

## Interferon responses in asthma

Viral respiratory tract infections are the most common triggers of acute asthma in children ^1^ and adults ^2^. Asthmatics, while not more prone to viral infections, have more severe symptoms and these infections trigger exacerbations of asthma and periods of worsened control ^3^. Given this observed susceptibility to the effects of viral infections, it has been proposed that asthmatics, by reasons of genetics or acquired factors, have a defect in antiviral immunity. Interferons (IFNs) play a central role in host immune responses to viral infections. The airway epithelium releases IFNs in response to viruses as part of the initial innate immune response to infection. They induce IFN-stimulated genes and lead to the production of antiviral proteins ^4^. IFNs consist of type I (IFN-α/-β), type II (IFN-γ), and type III (IFN-λ1, 2, and 3) ^5^. The impaired IFN response in asthmatics remains a contentious issue in the literature. We originally demonstrated ^6^ that rhinovirus-16 (RV-16) replication was increased in asthmatic bronchial epithelial cells (BECs) compared to healthy controls. This was due to an impairment in IFN-β, and when exogenous IFN-β was added to cultures, apoptosis was induced and viral replication reduced. This was later confirmed by Cakebread *et al.* ^7^, who showed that the expression of IFN-β-stimulated anti-viral genes was comparable in primary BECs from asthmatics and non-asthmatics. They suggested that IFN-β was able to reduce the expression of inflammatory cytokines such as CXCL-10, RANTES, and interleukin (IL)-6, suggesting that IFN-β may shorten the duration of inflammatory responses.

This was extended to type III IFN-λ by Contoli *et al.*, who found that asthmatic BECs were deficient in the induction of IFN-λ. This correlated with RV-induced asthma exacerbations and viral load in experimentally infected human volunteers ^5^. This deficiency in IFN-λ in primary BECs was again demonstrated by Parsons *et al.* They showed that this was due not to deficient expression of MDA5 and Toll-like receptor 3 (TLR3) but to an inability to later activate types I and III IFN immune responses to RV infection ^8^. These deficient innate responses were also demonstrated in children who were asthmatic regardless of their atopic status and in atopic patients without asthma by Baraldo *et al.* ^9^. However, other studies have found no change in IFN-β ^10^ or λ ^11^ in the asthmatic epithelium.

Investigating this process further, Kicic *et al.* recently found that BECs from asthmatic children had reduced IFN-β release and increased inflammatory cytokine production compared to healthy control cells. Exogenous IFN-β restored apoptosis, suppressed viral replication, and improved wound healing but did not reduce the heightened inflammatory cytokine production ^12^.

Deficient IFN responses and susceptibility to viral infection may be present in only some asthmatics, reflecting either airway pathology or the severity of disease. Simpson *et al.* ^13^ assessed the response of peripheral blood monocytes (PBMCs) to exposure to a single strain of RV from 86 adults with poorly controlled asthma despite treatment with moderate- to high-dose inhaled corticosteroids (ICS). They found that those who demonstrated impaired PBMC release of IFN-α to RV-1B had elevated airway neutrophils and were on the highest doses of ICS. Responses to infection then may vary depending on the characteristics of asthmatic inflammation, and there is the implication that treatment with high doses of ICS may increase susceptibility to viral infection in asthma. This is an area of great interest and could have significant implications for individually tailored IFN augmentation treatments.

## Type II immune inflammation and impaired interferon responses

Dysregulated type II immune responses are seen in a large proportion of asthmatics, especially those with childhood-onset disease. The mechanisms of impaired IFN responses and how they relate to the type II inflammatory responses typically seen in asthmatics is also of considerable interest in the current literature.

Contoli *et al.* ^14^ recently evaluated the effects of two archetypal type II immune cytokines, IL-4 and IL-13, on innate immune responses by BECs in response to RV infection. Human BECs were pre-treated with IL-4 and IL-13 for 24 hours and this led to impaired RV-16-induced activation of IRF3 and inhibited TLR3, resulting in a significant dose-dependent inhibition of IFN-β release 8 hours after infection. This resulted in increased RV replication in the cultures ^14^. This study demonstrates that a type II immune cytokine environment alone is sufficient to dampen innate immune responses in BECs.

Similar studies looking at double-stranded RNA (dsRNA) have had mixed findings. IL-13 was implicated to impair the production of dsRNA-induced IFN-λ expression from BECs ^15^. In contrast, Herbert *et al.* found that although IL-4 and IL-13 could promote pro-inflammatory cytokines by BECs in response to dsRNA, anti-viral host defenses in asthmatics were not impaired ^16^.

IL-4, IL-13, and RV infection were also able to induce suppressor of cytokine signaling 1 (SOCS1) expression. It was found that SOCS1 protein expression was increased in the airway epithelium from asthmatic adults measured in bronchial biopsy specimens, and the presence of SOCS1 correlated with a greater severity of atopy and airway hyper-responsiveness ^17^. The overexpression of SOCS1 in both primary BECs and a human epithelial cell line completely inhibited exogenous IFN-β-induced activation of both the IFN-β and the IFN-λ promoters. This overexpression also suppressed RV-induced IFN promoter activation in primary BECs and significantly increased IL-1β- and TNF-α-induced CXCL8. Therefore, increased levels of SOCS1 were shown to impair IFN induction in asthmatic patients through its nuclear localization, but this was specific for only antiviral immunity, as inflammatory mediators were not affected. This is a novel study revealing a possible mechanism for impaired IFN responses in asthmatics.

Jackson *et al.* showed that IL-33 and type II cytokines IL-4, IL-5, and IL-13 were induced by RV in the asthmatic airway *in vivo* and that these levels related to exacerbation severity. IL-33 was strongly induced by RV infection of primary bronchial cells *in vitro*. The supernatant from RV-infected human BECs was able to induce type II cytokine production by human T cells and type II innate lymphoid cells ^18^.

IL-33 has also been suggested as a link between innate immune activation and type II immune responses in asthma. In the airways of mild steroid-naïve asthmatics, IL-33 mRNA expression was also associated with increased TLR2 and TLR4 expression as well as allergic sensitization to house dust mite (HDM) and elevated levels of fractional exhaled nitric oxide (FeNO) ^19^. In mice, Tashiro *et al.* showed that IL-33 levels in lung homogenates were markedly increased in HDM-sensitized mice compared to control mice and that IL-33 levels were increased in mononuclear cells derived from the lungs of HDM-sensitized mice compared to controls ^20^.

Another emerging and important link between activation of the innate immune response in asthma and acquired type II immune responses that are driven by T helper type 2 (TH2) cells are innate lymphoid cells (ILCs). These are non-T, non-B lymphocytes that can be activated rapidly in response to external stimuli without the need for a specific antigen–antibody interaction. They are defined based on the types of cytokines they produce, those associated with asthma and allergic disease, release IL-5, IL-9, and IL-13, and are termed ILC2s. Activated by thymic stromal lymphopoietin (TSLP), IL-33, and IL-25, ILC2s release large amounts of type II cytokines leading to increased inflammation, recruitment of eosinophils to the airways, and airway hyperresponsiveness ^21– 23^.

The role for ILC2s in asthma is currently under intense investigation. In a pioneering study, Duerr *et al.* ^24^ demonstrated that type I IFNs reduced the capacity of IL-33-stimulated mouse and human ILC2s to proliferate and release type II cytokines IL-4, IL-5, IL-9, and IL-13 as well as the pro-inflammatory cytokines IL-6 and granulocyte macrophage colony-stimulating factor (GM-CSF). Type I IFN receptor-deficient mice had increased numbers of ILC2s and enhanced type II immune responses upon infection. Type I IFNs directly and negatively regulated mouse and human ILC2s dependent on the transcriptional activator IFN-stimulated gene factor 3 (ISGF3) that led to reduced type II cytokine production, cell proliferation, and increased cell death ^24^.

In turn, Lynch *et al.* used a mouse model using pneumonia virus of mice (PVM) and low-dose cockroach extract (CRE) to induce the features of allergic asthma and acute viral infection. CRE exposure during viral infection in early life induced IL-33, which suppressed IFN-α and IFN-λ production, increasing type II inflammation and the viral burden. This was shown to be through the downregulation of viperin and IRAK1 in plasmacytoid dendritic cells *in vivo* and *in vitro*, leading to a state of TLR7 hyper-responsiveness ^25^.

While overall very few in number, elevated numbers of ILC2s have been previously found in the peripheral blood of allergic asthmatics. Investigators from Shanghai set out to determine if IL-13 ^+^ ILC2s correlated with asthma severity ^26^. ILC2s were identified by flow cytometry as a distinct CD45hi IL-7Rα ^+^ CRTH2 ^+^ cell population from human PBMCs. The investigators found that the frequencies of ILC2s were increased in asthmatics (0.04±0.02%) compared to healthy subjects (0.025±0.011%) and the percentages of IL-13 ^+^ ILC2s were significantly higher in subjects with uncontrolled asthma (49.7±16.9%) and partly controlled groups (30.8±13.1%) compared to the well-controlled group (16.7±5.9%) and healthy controls (18.7±8.7%). Effective clinical treatment of uncontrolled IL-13 ^+^ ILC2-positive asthmatics resulted in dynamic modulation of IL-13 ^+^ ILC2 levels back to baseline. ILC2s were strongly stimulated by IL-25/-33 and, interestingly, when the investigators pretreated cells with dexamethasone, they found ILC2s were more resistant to suppression than TH2 cells *in vitro* ^26^.

**Figure 1.**
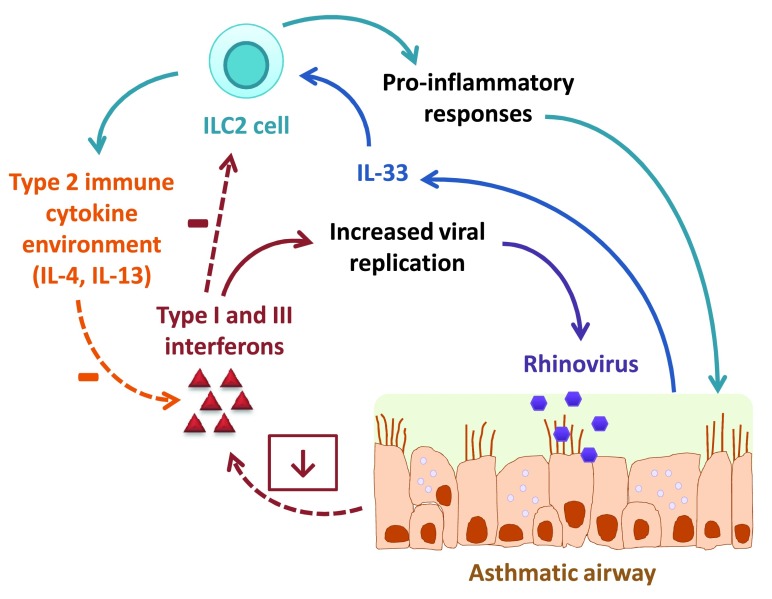
Interactions between the type II immune cytokine environment and interferon responses in the asthmatic airway during rhinovirus infection. The asthmatic airway is suggested to be deficient in the production of interferons in response to rhinovirus infection. This contributes to increased viral replication. The type II immune cytokines interleukin (IL)-4 and IL-13 have been shown to dampen interferon responses, possibly through the induction of suppressor of cytokine signaling 1 (SOCS1). IL-33 is induced by rhinovirus infection and activates type 2 innate lymphoid cells (ILC2s), which in turn release type II cytokines and pro-inflammatory cytokines such as IL-6 and granulocyte macrophage colony-stimulating factor (GM-CSF). Recent findings demonstrate the ability of type I interferons to suppress IL-33-stimulated ILC2 responses.

These studies provide exciting links between innate anti-viral responses and the type II cytokine inflammatory conditions seen in asthma and are summarized in Figure 1. If a type II cytokine environment suppresses IFN responses, and IFN responses are able to suppress ILC2s, then in asthma there could be a positive cycle feeding into an exaggerated pro-inflammatory cytokine response, as well as a perpetuation of viral replication. This could provide insights into what happens during a viral infection and exacerbations leading to worsened asthmatic inflammation.

## The role of the airway epithelium in asthma

BECs have been recognized as having an important contribution to the innate immune response but are thought to have a limited contribution to adaptive immune responses. Typically, TH1-derived IFN-γ inhibits TH2 cell differentiation, whereas TH2-derived IL-4 promotes TH2 differentiation. It is unknown if airway epithelial cells have the same TH1/TH2 programs as lymphocytes. BECs recognize IL-4 as well as express the receptor for its structural homolog, IL-13. Airway epithelial cells also express IFN-γ receptors. Zissler *et al.* exposed primary BECs exposed to IL-4 or IFN and then performed genome-wide transcriptome analysis. The study demonstrated an antagonistic regulation pattern of IL-4 and IFN-γ, translating the TH1/TH2 antagonism directly to epithelial gene regulation. IL-4 and IFN-γ induced separate transcription factor hubs or clusters, which are present in antagonistically and polarized gene regulation networks. Furthermore, the IL-4-dependent induction of IL-24 observed in rhinitis patients was downregulated by IFN-γ; therefore, IL-24 represents a potential unique biomarker of allergic inflammation and a TH2-polarized epithelium ^27^.

## Early life asthma

The prevention of asthma or modifying the risk of its development *in utero* would clearly be enormously beneficial to society and for those suffering from asthma throughout their lives. Vitamin D has long been implicated in the pathogenesis of asthma and immunity, and two important trials sought to determine if vitamin D3 supplementation could influence the development of asthma in early childhood. The first trial, which was based in Europe, recruited 623 women at 24 weeks and followed 581 children to age 3 ^28^. Women were randomized to receive the usual supplement of 400 IU or vitamin D at 2400 IU, with the primary aim being that supplementation would reduce persistent wheeze at age 3 years. The intervention arm had 16% develop persistent wheeze versus 20% of controls, which was not significantly different, though other secondary outcomes such as troublesome episodes of lung problems were reduced. The second trial reported in the same journal was a larger US study in three centers and followed 806 children to age 3 but recruited women whose children were at high risk of asthma ^29^. The intervention arm received high-dose (4000 IU) vitamin D and the control arm received 400 IU. With the selection of a higher risk group, more children (218/806) developed persistent wheeze, and, while there was an absolute reduction in persistent wheeze by 6%, this also failed to reach significance. Interestingly, most of the effect appeared to occur in the first 1–2 years and then may have waned. In both studies, those who received extra supplementation were more likely to be vitamin D sufficient. The results seen in both trials were similar, and it is possible that the trials were underpowered to detect such a complex outcome, though as a consequence the case that vitamin D supplementation will modify asthma risk remains unproven.

The risk of developing early life asthma is complex and it has been known for some time that this risk is modified by gender, with a heightened risk in boys that switches to girls at puberty ^30^. The complexity of early life asthma was illustrated by a cross-sectional US epidemiological survey that further emphasized the importance of gender ^31^. In 1623 singleton live births that were followed by questionnaire at 6 months and then yearly until age 9, the investigators found 12.7% were early transient wheezers and 13.1% became persistent wheezers. Compared with never/infrequent wheeze, maternal asthma, infant bronchiolitis, and atopic dermatitis were associated with persistent wheeze in both sexes, but paternal asthma was associated with persistent wheeze in boys only (odds ratio [OR] 4.27; 95% confidence interval [CI] 2.33–7.83), while being black or Hispanic was a predictor for girls only. The strong association between paternal asthma and risk in boys is certainly emphasized as well as confirming the role of other previously described environmental and social factors. These studies emphasize that for boys, paternal inherited factors appear to play an important role in developing persistent asthma and what those factors are should be further investigated.

## Sublingual dust mite immunotherapy and asthma

The close association between asthma and allergy to dust mites in childhood has led to numerous interventions to reduce the impact of this exposure on asthma. The advent of sublingual immunotherapy has revised academic interest in desensitization, opening this up as a therapy to more individuals with less disruption and potentially less risk compared to subcutaneously administered protocols of desensitization. Virchow *et al.* ^32^ took 834 adults sensitized to HDM with asthma that was not well controlled on ICS alone. Subjects were randomized to receive either placebo or two doses of sublingual HDM; the primary outcome was a reduction in time to first moderate to severe exacerbation. Both of the doses reduced the risk of asthma exacerbation (hazard ratio 0.72; 95% CI 0.52–0.99), though there were no changes in asthma control scores or quality of life. While the effect seen was modest, it does demonstrate that chronic allergen exposure plays a direct role in exacerbation risk and, if reduced, modifies asthma.

## Modifying exacerbations of asthma by controlling allergic disease

Exacerbations of asthma in children continue to occur despite treatment and are more likely in those with more severe disease and a history of previous exacerbations ^33^. An interesting phenomenon that has been seen with these exacerbations has been a rise that occurs when children first return to school from their summer holidays that has been attributed to the combined effects of allergen exposure and the exacerbation triggered by a viral infection. An intervention that disrupts this interaction therefore could have an important effect on improving asthma care. This was proposed by Teach *et al.* ^34^ when they selected atopic children aged 6–17 years who had a previous history of asthma exacerbation despite regular treatment with ICS at a dose of at least 200 mcg/day fluticasone equivalence. Before the fall seasons of 2012 and 2013, 727 children were enrolled, 513 were randomized, and 478 were analyzed. Subjects received omalizumab (a monoclonal antibody against immunoglobulin E [IgE] that has been shown to reduce asthma exacerbation frequency), a boost in their ICS dose, or placebo. The fall in exacerbation rate was significantly lower in the omalizumab versus placebo arms (11.3% versus 21.0%; OR 0.48; 95% CI 0.25–0.92), but there was no significant difference between omalizumab and the ICS boost (8.4% versus 11.1%; OR 0.73; 95% CI 0.33–1.64). In a pre-specified subgroup analysis, among participants with an exacerbation during the run-in phase, omalizumab was significantly more efficacious than both placebo (6.4% versus 36.3%; OR 0.12; 95% CI 0.02–0.64) and ICS boost (2.0% versus 27.8%; OR 0.05; 95% CI 0.002–0.98). Omalizumab was also more effective in those who were defined as having more severe disease requiring treatment during the run-in phase. In an additional subgroup analysis of 87 subjects, PBMCs were taken before and after changes in treatment and the immune response in terms of IFN-α response to RV was assessed. Omalizumab improved IFN-α responses to RV, and within the omalizumab group, greater IFN-α increases were associated with fewer exacerbations (OR 0.14; 95% CI 0.01–0.88). This trial for the first time accurately identified that children with more severe disease – including a history of serious exacerbations despite regular ICS treatment – will benefit from more intense treatment of their asthma, directly reducing their susceptibility to virus-induced exacerbations of asthma.

## The consequences of childhood asthma for lung health throughout life

One of the most revealing studies to come from 2016 was the longitudinal follow-up of the childhood asthma management project (CAMP) cohort. This original study took 1041 children aged 5–12 years with chronic mild to moderate asthma and treated them for 5 years with inhaled budesonide, nedocromil, or placebo ^35^. The interventions at the time failed to influence lung function growth. A paper by McGeachie *et al.* ^36^ now reports on the pattern of lung function 13 years after the trial concluded in 85% of the cohort. In persons without lung disease, forced expiratory volume in 1 second (FEV1) reaches its maximal level in late adolescence or early adulthood (usually around 20 years of age) and remains stable for several years, a period known as the plateau of lung function, before gradually declining, usually after 25 years ^37^. The authors found in this group with mild to moderate asthma that 75% had abnormal lung growth and decline. Impaired lung function at enrollment and male sex were the most significant predictors of abnormal longitudinal patterns of lung function growth and decline. A pattern of reduced growth was evident early in childhood and was found to persist into early adulthood; 52% of patients had early lung function decline. By the age of 26 years, 11% of the cohort met the post-bronchodilator spirometric definition of chronic obstructive pulmonary disease (COPD). This study clearly emphasizes the enormous long-term effect childhood asthma can have on lung function throughout life. It makes the case that identifying and following those with abnormal spirometric trajectories may be important to identify those at risk of adult lung disease, though it is sobering that we still do not have an intervention that has been shown to alter these outcomes.

## Better drug delivery improves asthma outcomes

The advantage in asthma of using medications that are inhaled is that the drug can be delivered to the affected airway, the site of disease, while minimizing off-target side effects. In asthma, inflammation is present throughout the airways, including peripheral ones or small airways <2 mm in diameter ^38^, and these airways contribute to both inflammation and airway resistance ^39^. Therefore, systems that more effectively deliver therapy throughout the airways, especially ICS, may be more effective. Preparations of extra-fine hydrofluoroalkanes (HFAs) deliver ICS particles <2 μm (HFA-ciclesonide and HFA-beclomethasone) with lung deposition of >50% compared to CFC fluticasone or dry powdered fluticasone where the size is >2 μm in size and lung deposition is between 10 and 15% ^40^. In addition, a small-particle aerosol that remains suspended in the air for longer should provide an advantage for dis-coordinated patients by improving lung deposition of the drug and reducing the dependence on correct inhaler technique. With these advantages in mind, Hodgson *et al.* ^41^ set out to determine in 30 asthmatics with refractory eosinophilia if the addition of extra-fine ciclesonide to the usual ICS regime and/or regime of oral corticosteroids (OCS) would suppress airway eosinophils. They selected subjects with persistent airway eosinophilia (sputum eosinophils >3% or peripheral blood eosinophils >0.4 × 10 ^9^/ml) and demonstrated that a short course of prednisone 30 mg/day would normalize exhaled nitric oxide (<25 ppB) and improve FEV1 and asthma control. They then added either ciclesonide 320 mcg/day or placebo to the usual ICS regime (median dose of beclomethasone equivalent 1600 mcg/day) or low-dose regular OCS (a third remained on OCS). Ciclesonide appeared to be better at maintaining sputum eosinophils <3%, but this did not demonstrate significance until those subjects who had (against protocol) reduced their OCS were excluded; the median sputum eosinophils compared between the groups were 1.4% ciclesonide versus 4.5% controls, p=0.028.

A direct comparison was made of the use of ICS extra-fine ciclesonide (dose 160–320 mcg) with the use of fluticasone (500 mcg/day) in 1390 milder asthmatics aged 12–60 years ^42^. Despite the lower dose of ICS, the ciclesonide arm demonstrated fewer acute exacerbations, with an adjusted rate ratio of 0.59 (95% CI 0.47–0.73).

Finally, there was the example of applying technology to deliver corticosteroids to the airways more effectively. Vogelmeier *et al.* ^43^ tested the application of a new computer-controlled air compressor (AKITA) to deliver budesonide solution to a group (n=199) with severe, GINA step 5 asthma requiring regular OCS. There were four arms: air-interfaced culture (AIC) budesonide 1 mg, AIC budesonide 0.5 mg, budesonide 1 mg delivered by standard nebulizer, and placebo. In the run-in phase, subjects were able to have their OCS dose reduced by 50%. Following treatment, both AIC arms demonstrated improved FEV1 compared to standard nebulized budesonide and placebo, with the AIC 1 mg arm demonstrating a significant reduction in exacerbations at 7% compared to standard budesonide at 22% and placebo at 19%.

## Biological agents and asthma

Biologic agents represent a major innovation in the treatment of chronic immune-mediated disease and have led to important advances in the management of rheumatoid arthritis, inflammatory bowel disease, and, since the advent of omalizumab, severe asthma. Dupilumab is a monoclonal antibody that binds to the IL-4 receptor-α and has the ability to block the action of both IL-4 and IL-13. It has demonstrated promising efficacy in the treatment of allergic rhinitis and nasal polyposis, eczema, and asthma with elevated blood eosinophils ^44^. In their publication, however, Wenzel *et al.* ^45^ did not confine treatment to those with persistent eosinophilia but selected 729 subjects with persistent symptoms despite treatment with medium- to high-dose ICS and long-acting beta agonists (LABAs). Patients were randomly assigned (1:1:1:1:1) to receive subcutaneous dupilumab 200 mg or 300 mg every 2 weeks or every 4 weeks, or placebo, over a 24-week period. The primary endpoint was change from baseline at week 12 in FEV1, and subgroup analysis was done based on blood eosinophils < or > 300/μL. The best effect was seen with biweekly dosing and little difference was seen between 200 mg and 300 mg doses. The effect size appeared slightly greater in those with elevated blood eosinophils, but significant improvements were seen in all patients. The greatest change in FEV1 compared with placebo was observed at week 12 with doses every 2 weeks in the 300 mg group (mean change 0.39 L [SE 0.05]; mean difference 0.21 [95% CI 0.06–0.36; p=0.0063]) and in the 200 mg group (mean change 0.43 L [SE 0.05]; mean difference 0.26 [0.11–0.40; p=0.0008]) compared with placebo (0.18 L [SE 0.05]). These changes were sustained to week 24. In addition, subjects demonstrated reductions in annualized rates of exacerbation in the overall population (70–70.5%), the subgroup with at least 300 eosinophils/μL (71.2–80.7%), and the subgroup with fewer than 300 eosinophils/μL (59.9–67.6%). The most common adverse events with dupilumab compared with placebo were upper respiratory tract infections (33–41% versus 35%) and injection-site reactions (13–26% versus 13%). Dupilumab offers perhaps the most promise of any of the current biological agents proposed for managing severe asthma. It appears to have favorable effects on important co-morbidities such as chronic sinusitis, nasal polyposis, and atopic dermatitis. In comparison, omalizumab is confined to only those with atopic asthma, where an improvement in FEV1 has not been shown and the reduction in exacerbation rates is only 25% ^46^. Monoclonal antibodies to IL-5 appear to have their efficacy limited to those with at least 150 eosinophils/μL, and in these individuals the reduction in exacerbation rate is in the order of only 47% ^46^. In addition, the fact that dupilumab – an agent that blocks the classic type II immune IL-4/-13 pathway in asthma – demonstrated efficacy in those with and without eosinophilia raises the possibility that our ability to identify when type II immune mechanisms are active with biomarkers remains imperfectly understood.

A second-generation monoclonal antibody to IgE, ligelizumab, has been developed and this demonstrates significantly greater *in vitro* binding affinity than does omalizumab ^47^. In a small proof-of-concept trial, 37 mild atopic asthmatics were randomized to receive ligelizumab, omalizumab, or placebo ^47^. Ligelizumab attenuated the effect of allergen challenge threefold more effectively than did omalizumab and 16-fold better than did placebo. It also more effectively suppressed skin test reactivity to sensitized allergens, which peaked after 18 weeks of treatment.

## Targeting non-type II immune responses in asthma

Most of the emerging therapies for asthma target type II immune mechanisms. However, at best, abnormalities in type II immune responses occur in only 50% of adults with allergic asthma. As new therapeutics start to bring type II immune-mediated asthma under control, researchers need to focus greater efforts on defining the mechanisms of disease in these other patients to realize better outcomes ^48^.

One important group with difficult-to-control asthma is older adults with obesity. Obesity is common in asthma and is associated with systemic inflammation and metabolic dysfunction, such as insulin resistance and hypertension. The presence of low-grade systemic inflammation occurs in only a subgroup with obesity, but it predicts the presence of these other important comorbidities that make up the metabolic syndrome ^49^. Researchers for the Severe Asthma Research Program and the University of California San Francisco combined data and an analysis of stored samples from 387 well-characterized subjects with severe asthma and 249 milder asthmatics as well as 93 healthy controls. Using a cutoff level of serum IL-6 at 3.1 pg/mL to define systemic inflammation, they divided the subjects into IL-6 high and low groups ^50^. The IL-6 high group was more common in the cohort with severe asthma and had lower FEV1, worsened asthma control, and more frequent exacerbations. They were more likely to be female, have a higher body mass index (BMI), and be more were hypertensive. However, high IL-6 was not related to either blood eosinophils or exhaled nitric oxide. While 86% of those with high IL-6 were obese, only 62% of obese asthmatics had elevated serum IL-6. Using a BMI of >30 as a cutoff, those with elevated IL-6 and obesity had the lowest FEV1, had more exacerbations, and were more likely to have features of metabolic syndrome. This work certainly identifies serum IL-6 as an important biomarker in severe asthma and links it to obesity and metabolic syndrome.

In light of the recent evidence of serum IL-6 playing a role in difficult asthma and metabolic syndrome, a small trial by Franca-Pinta *et al.* ^51^ offers further insight and strategies to intervene. The authors took 58 asthmatics and recruited them to undergo a regime of aerobic exercise training or a sham intervention for 12 weeks. At the end of 12 weeks, the exercise group demonstrated reduced bronchial hyper-reactivity as well as a reduction in serum IL-6. Interestingly, though, there was no change in sputum cell counts or exhaled nitric oxide.

## The mechanisms of disease in severe asthma differ from mild to moderate disease

Severe asthma is known to behave in a clinically distinct manner to mild and moderate disease, which is characterized by type II immune pathology and corticosteroid responsiveness. In contrast, severe asthma is inherently corticosteroid resistant and appears to be associated with more complex airway pathology ^52, 53^. Further light has been shed on these mechanisms by Raundhal *et al.* ^54^. They examined bronchial lavage from severe and mild/moderate asthma, finding that the majority of T cells were CD4. In severe asthma, there were more CD4 cells that expressed IFN-γ, TH17 cells were also more numerous, while both groups had low numbers of TH2 cells. The severe asthma immune response was then modeled in mice to determine the functional significance of a higher TH1 response. They found that it was IFN-γ, and not IL-17, that mediated bronchial reactivity. However, IL-17 led to neutrophil accumulation in the airways. Both in humans and in mice, the expression of secretory leukocyte protease inhibitor (SLPI) was downregulated in epithelial cells as a consequence of IFN-γ exposure. As SLPI can inhibit bronchial reactivity, this may provide an explanation for the poor lung function seen in severe asthma. When SLPI expression was enhanced in the mice, this reduced bronchial reactivity and augmented the response to corticosteroids. This work paints a very complex picture of severe asthma, where non-type II immune pathology and IFN-γ/SLPI drive bronchial reactivity through as-yet-unknown mechanisms and IL-17 may play a secondary role in recruiting neutrophils but where the presence of TH2 cells, albeit in low numbers, could still be important.

## Conclusions

The last 12 months have once again seen significant activity in the area of asthma research, resulting in improved understanding and new insights with regard to its management. Important research is now emerging that demonstrates crucial links between the innate immune response and traditional type II acquired immune responses in asthma. We would predict this to be one of the next areas to emerge where the development of targeted therapies could lead to important new treatments. As this area is crucial in the pathogenesis of asthma in childhood and the interaction of viral infection and allergic sensitization, it may hold hope for the development of treatments that will specifically target acute asthma and potentially influence the progression of early childhood disease.

Clinical trials of biological agents are likely to continue to evolve, though at this stage they are still the most efficacious in those with severe disease and those with either allergic disease or dysregulated type II immune responses. It is possible that in the near future, if they are applied in their current forms to early disease, they may modify the disease course in asthma.

A major deficiency, however, remains in the lack of understanding of the mechanisms of disease that exist in those asthmatics with non-type II immune responses. They make up an important and growing body of asthmatics with a high disease burden. They are associated with other chronic diseases, where the processes underlying asthma may overlap and impact on these processes as well. They are known to respond relatively poorly to current asthma treatments, and while the mechanisms driving disease in these cases remain unclear and until a better understanding of these processes are made, more effective treatments will remain elusive.
